# Solution Combustion Synthesis of High-Performance Nano-LiFePO_4_/C Cathode Material from Cost-Effective Mixed Fuels

**DOI:** 10.3390/ma16227155

**Published:** 2023-11-14

**Authors:** Haozhi Duan, Dehai Meng, Shuxia Yuan

**Affiliations:** 1 National Engineering Laboratory for Carbon Fiber Technology, Institute of Coal Chemistry, Chinese Academy of Sciences, 27 Taoyuan South Road, Taiyuan 030001, China; duanhaozhi@sxicc.ac.cn (H.D.); mengdh@sxicc.ac.cn (D.M.); 2 CAS Key Laboratory of Carbon Materials, Institute of Coal Chemistry, Chinese Academy of Sciences, 27 Taoyuan South Road, Taiyuan 030001, China

**Keywords:** LiFePO_4_, cathode materials, solution combustion synthesis, electrochemical performance, mixed fuels

## Abstract

Solution combustion synthesis (SCS) is considered as an efficient and energy-saving method for preparing LiFePO_4_/C composite material with the nanostructure (Nano-LiFePO_4_/C). In this study, Nano-LiFePO_4_/C cathode material was prepared using SCS using a cost-effective combination of urea and sorbitol as mixed fuels. The effect of mixed fuels on combustion behavior and microstructure as well as on electrochemical performance was studied using XRD, BET, SEM, TEM, and electrochemical characterization methods. Multiple characterization results indicated that the maximum temperature (T_m_) and particle size were influenced by the usage of urea and sorbitol. The sample derived under optimum conditions exhibits a mesoporous nanostructure with a large surface specific area and attractive electrochemical performance with a discharge capacity of 153.5 mAh/g at 0.1 C, which shows strong potential for commercial applications in the future.

## 1. Introduction

Rechargeable lithium-ion batteries (LIBs) have rapidly integrated into various facets of our daily lives over the past decades, particularly as portable energy storage solutions, owing to their high energy density and extended cycle life. Consequently, extensive research has been dedicated to LIBs to address the growing demand for enhanced lithium utilization and specific energy density [1]. Since the cathode materials employed in LIBs significantly underpin their performance, a large amount of research has concentrated on advanced cathode materials [2]. Since it was first introduced in 1997, olivine-structured lithium iron phosphate (LiFePO_4_) has emerged as one of the most widely used cathode materials employed in rechargeable lithium-ion batteries [3]. It has been extensively studied due to its affordability, abundant raw material supply, a high theoretical capacity of 170 mAh/g, superior cycle performance, and remarkable stability at elevated temperatures [4]. However, it encounters the challenge of low intrinsic conductivity for both lithium ions and electrons, leading to its poor performance at high rates and low temperatures [5]. To address this limitation, two strategies have been explored extensively in recent years. Firstly, particle nanosizing has been implemented to reduce the diffusion distance, enhancing the efficiency of ion and electron transfer. Additionally, surface-coated carbon has been widely recognized as an effective approach to extend the surface area available for the interfacial transfer of conductive particles and, thus, improve the low electronic conductivity of LiFePO_4_ [6,7].

In recent years, carbon coated LiFePO_4_ with the nanostructure (Nano-LiFePO_4_/C) has been prepared using various physical and chemical synthesis routes. The physical solid-state method, which has developed over a few decades, stands out as the most prevalent technique for producing Nano-LiFePO_4_/C [8,9,10,11]. However, the solid-state approach is associated with several shortcomings: (1) The preparation process includes a series of complex procedures, including high-energy milling, spray drying, calcination, and grinding. Not only does this result in costly equipment requirements, but it also leads to a huge waste of energy and time. (2) Given that the raw materials are a heterogeneous mixture of solids, it makes it difficult to achieve uniform composition [12]. (3) The particle size is inherently related to the size of the initial raw materials, and in the solid-state method the final product often yields particles larger than 500 nm. These limitations significantly hamper its application in high-rate and low-temperature batteries. Chemical methods such as the hydrothermal/solvothermal method, the sol–gel method, the spray pyrolysis method, high mixing continuous rotating reactor technology (HMCRR), and the solution combustion method were notable for their high homogeneity and small particle size (~100 nm), which is difficult to achieve in the solid-state method [11,13,14]. Among these solution synthesis routes, solution combustion synthesis (SCS) stands out as a novel and efficient approach for the preparation of nanoscale materials. In this method, a strongly exothermic chemical reaction between an oxidizer (generally metal nitrites) and organic fuels in an aqueous solution is utilized. After further calcination with a carbon source, such as glucose, ultrafine Nano-LiFePO_4_/C nanoparticles were produced [15]. Solution combustion synthesis (SCS) possesses several noteworthy characteristics: (1) The SCS reaction finishes in a short time and a portion of the heat arises from the self-propagating reaction, rendering it an energy-efficient and time-saving methodology. (2) The raw materials are intimately mixed in an aqueous solution at the molecular level, ensuring a high level of homogeneity in the resulting product. (3) SCS yields products characterized by a high specific surface area and a finely structured nanoarchitecture, achieved through the generation of large volumes of gases. These attributes significantly enhance the electrochemical performance of LiFePO_4_/C [16].

In previous studies, Nano-LiFePO_4_/C has been synthesized with a single fuel such as sucrose, glycine, urea, L-Lysine, or glucose [15,17,18,19,20]. However, the electrochemical performance of the obtained LiFePO_4_/C was far from satisfactory due to the large particle size and particle agglomeration. Recently, a mixture of organic fuels with different functions has been verified to be useful in modifying the performance of LiFePO_4_/C using SCS. Nano-LiFePO_4_/C prepared with CTAB-based mixed fuels showed an ultrafine structure and a specific capacity of 137 mAh/g at 0.1 C [21,22]. In the mixed fuels, CTAB played the role of a cationic surfactant and performed as a soft template during the gelation process, promoting control over the final combusted products, while citric acid or glycine were used for gelation, leading to less agglomeration and high homogeneity. However, fuels such as CTAB and glycine are expensive materials, which makes it difficult to achieve large-scale fabrication [23,24].

In this study, inexpensive and easily accessible urea–sorbitol mixed fuels have been developed to prepare Nano-LiFePO_4_/C using SCS, where sorbitol was used as a complexing agent for Fe^3+^ to avoid precipitation and decomposable urea served to prevent the agglomeration of particles. The effect of mixed fuels on the combustion behavior, microstructure, and electrochemical performance of Nano-LiFePO_4_/C was studied in detail.

## 2. Experimental Procedure

### 2.1. Synthesis of Nano-LiFePO_4_/C Cathode Material

**Figure 1:**
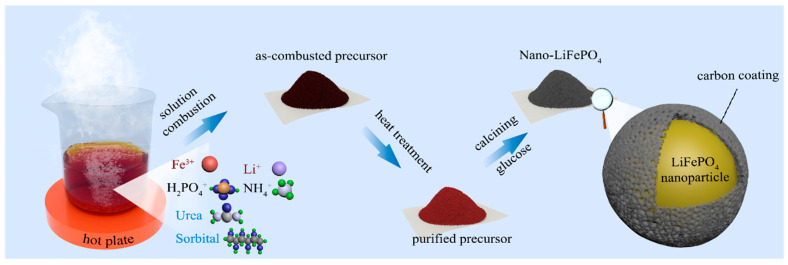
Schematic illustration of preparation process of Nano-LiFePO_4_ using SCS.

Fe (NO_3_)_3_·9H_2_O, LiNO_3_, NH_4_H_2_PO_4_, urea, and sorbitol with A.R. grade were provided by the Aladdin company (Shanghai, China). As shown in Figure 1, 24 mmol Fe (NO_3_)_3_·9H_2_O, 25 mmol LiNO_3_, 24 mmol NH_4_H_2_PO_4_, 24*x* mmol urea, and 24*y* mmol sorbitol were dissolved in 20 mL deionized water. The mixture was heated at 80 °C until uniform and transparent solution was formed. The solution was subsequently transferred to a preheated hot plant (at 350 °C) within a fume hood in an ambient air environment. Within a few seconds, the solution was ignited resulting in the release of large amounts of gases yielding slurry precursor powders. The precursor powders were heated to 500 °C in air for 15 min to remove residuals from mixed fuels [25]. Then, the obtained powders were milled by hand and mixed with 20 wt% glucose as carbon source. Then, the mixture was calcined at 750 °C for 6 h in N_2_ atmosphere in a tube furnace with a heating rate of 4 °C/min to obtain carbon coated Nano-LiFePO_4_/C. The amount of raw material is calculated according to the following reaction (Equations (1) and (2)), where Fe (NO_3_)_3_·9H_2_O, LiNO_3_ was oxidizer and urea and sorbitol were acting as fuels. In this reaction, *φ* ≥ 1 means fuel rich while *φ* < 1 means fuel lean. For easy identification, the samples with different amounts of urea and sorbitol were coded as U*_x_*S*_y_*. Except for U_1.6_S_0_, all SCS was carried out with sufficient fuels (*φ* ≥ 1). The commercial LiFePO_4_/C was purchased from BTR Company (Shenzhen, China).

(1) $$\begin{array}{l} {\mathrm{LiNO}}_{3}+\mathrm{Fe}{\left({\mathrm{NO}}_{3}\right)}_{3}\cdot 9H_{2}{O+\mathrm{NH}}_{4}H_{2}{\mathrm{PO}}_{4}{+\mathrm{xCH}}_{4}N_{2}O\\ {+\mathrm{yC}}_{6}H_{14}O_{6}+\frac{9}{2}(\varphi -1)O_{2}\to LiFePO_{4}+(x+6y)CO_{2}+\\ (2x+7y+12)H_{2}O+(x+\frac{5}{2})N_{2} \end{array}$$

(2) $$\varphi =\frac{3x+13y}{9}$$

### 2.2. Material’s Characterization

The combustion behavior of precursor (dried at 80 °C) was analyzed using thermo gravimetric analysis (TGA, Q600, TA Instrument, Eden Prairie, MN, USA) in air atmosphere at a heating rate of 10 °C/min. Carbon content test was conducted using carbon and sulfur combined tester (CS844, Leco, St. Joseph, MI, USA). The temperature of precursor solution during combustion was tested using a thermocouple (K-05, SUMA Instrument, Taizhou, China) placed in the solution. The structural properties were analyzed using X-ray powder diffraction (XRD, DX-27, HAOYUAN Instrument, Dandong, China). The powders were scanned using CuKa radiation (k = 1.54060 Å) with a scan rate of 0.06/1 s at room temperature. Rietveld refinement was performed using the GSAS (II) (General Structure Analysis System) program to determine the crystal structure parameters. The specific surface area and pore size distribution were examined using N_2_ adsorption apparatus (TriStar II 2020, Micromeritics, Norcross, GA, USA). The structure, particle morphology, and element distribution were investigated using scanning electron microscopy (SEM, JSM7900, JEOL, Akishima, Japan) equipped with an energy dispersive X-ray spectroscope (EDS, Plano, TX, USA). The structure and thickness of carbon coating were assessed using a high-resolution transmission electron microscope (HRTEM, G2F20S-Twin, Tecnai, FEI, Inc., Valley City, ND, USA). The electronic conductivity of the samples was measured using a four-probe powder tester (FT-301B, Ningbo Recke Micro Intelligent Technology Co., Ltd., Ningbo, China) at a pressure of 20 MPa. The particle size distribution was measured using Nano Measurer 1.2 software and calculated using Origin 2023 software.

### 2.3. Preparation of Electrode Sheet

The Nano-LiFePO_4_/C cathode was prepared as a slurry by mixing the active material, Ketjen black, and poly (vinyl difluoride) (PVDF) binder with a weight ratio of 90:5:5 using an agate mortar and pestle. In the mixing process, powders were well dispersed in N-methyl pyrrolidone (NMP) as solvent. Then, the as-prepared slurry was coated onto well cleaned aluminum foil (purchased from Canrd Company, Dongguan, China, thickness ~ 17 μm) acting as a current collector using a blade-coater, and dried at 60 °C in vacuum for 12 h to remove the NMP solvent. The loading of active materials at each cathode was approximately 1.5–2.5 mg/cm^2^. Then, the dried slurry loaded electrode sheet was cut into discs with a diameter of 10 mm.

### 2.4. Preparation of Coin-Type Cells

The coin-type cells (CR2032) were assembled in the argon-filled glove box with Nano-LiFePO_4_/C electrode working as cathode, lithium foil (15.6 mm × 0.45 mm) serving as anode, and commercial Celgard 2500 was applied as membrane. The electrolyte was made by dissolving 1 M LiPF_6_ in a mixture of ethylene carbonate and dimethyl carbonate (1:1 in *v*/*v*) as solvent.

### 2.5. Electrochemical Measurements

The rate charge/discharge tests in the voltage range of 2.0–3.8V vs. Li/Li^+^ electrode were carried out on LANHE CT-3002A (Wuhan land Co. Ltd., Wuhan, China). The EIS was analyzed using a CHI660E electrochemical workstation (CH instruments, Austin, TX, USA) with frequency ranging from 0.1 Hz to 100 kHz and an AC amplitude of 5 mV.

## 3. Results and Discussion

**Figure 2:**
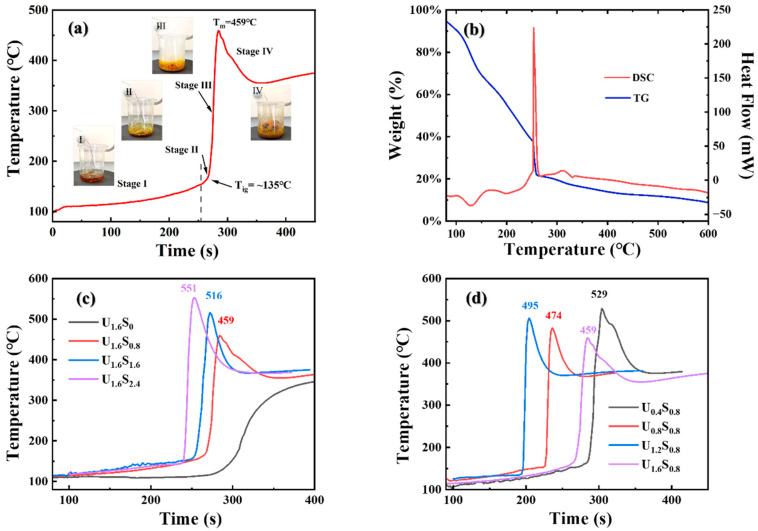
(**a**) Time–temperature profile of SCS process of U_1.6_S_0.8_. The insert shows photos of precursor at different stages. (**b**) TG/DSC curves at a heating rate of 10 °C/min. (**c**) Time–temperature curves of SCS process of U_1.6_S*_y_*. (**d**) Time–temperature curves of SCS process of U*_x_*S_0.8_.

Figure 2a shows the typical temperature profile of the mixed solution during the solution combustion process, which shows the characteristics of the volume combustion mode [26]. The entire solution was initially heated uniformly with the evaporation of free and part of bond water resulting in a mild temperature change (Stage Ⅰ). Subsequently, the temperature increased at a higher rate following serious bumping (Stage Ⅱ). When the temperature reached ignition temperature (*T_ig_*~135 °C), it increased sharply to the maximum temperature (*T_m_* = 459 °C) and was accompanied by the release of large amounts of gases (N_x_O, CO_2_, H_2_O, and NH_3_) and rapid volume expansion (Stage III), but no flame was observed. After the cooling stage (Stage Ⅳ), foamy as-combusted samples were obtained. Figure 2b presents the TG/DSC curves of the mixture during solution combustion. The evaporation of water and the decomposition of urea and Fe (NO_3_)_3_ should be responsible for a constant loss in weight of about 60% and a series of endothermic peaks on the DTA curve before 240 °C. Accompanied with a strong exothermic reaction between the oxidizer and the organic fuels, the mixture was rapidly dried. At the same time, a sudden mass loss of around 20 wt% happened at around 246 °C, with large amounts of gas and thermal energy released. At the same time, the temperature of the mixture reached the maximum. After the combustion reaction, weight loss still continued owing to the slow decomposition of residual organic fuels until all fuels were consumed [27]. Hence, the rapid change in temperature could be considered as an indication of the occurrence of the solution combustion reaction. Time–temperature curves for the SCS of the solution using varying amounts of urea and sorbitol were shown in Figure 2c,d. It is evident that the fuel to oxidant ratio and the composition of the mixed fuels influence the maximum temperature as well as the combustion behavior. Notably, in the sample only containing urea as fuel (U_1.6_S_0_), no temperature peak was observed, indicating a combustion reaction had not occurred with single urea under the fuel-lean condition. According to combustion theory, *T_m_* was determined using the initial temperature (*T*_0_), the heat of combustion (*Q_c_*), and the heat dissipation (*Q_d_*):

(3) $$T_{m}=T_{0}+\frac{Q_{c}-Q_{d}}{{\left(C_{p}\right)}_{product}}$$

where *C_p_* is the heat capacity at a constant pressure. In the same reaction system, larger *Q_c_* and smaller *Q_d_* will lead to a higher *T_m_* [15]. Except for U_1.6_S_0_, samples show similar time–temperature curves. Regarding U_1.6_S*_y_*, the *T_m_* increased with an increasing amount of sorbitol, suggesting a more vigorous reaction (larger *Q_c_*) was happened. Meanwhile, the *T_m_* tended to decrease with the amount of urea as more heat was taken by the endothermic decomposition reaction of the urea (larger *Q_d_*).

**Figure 3:**
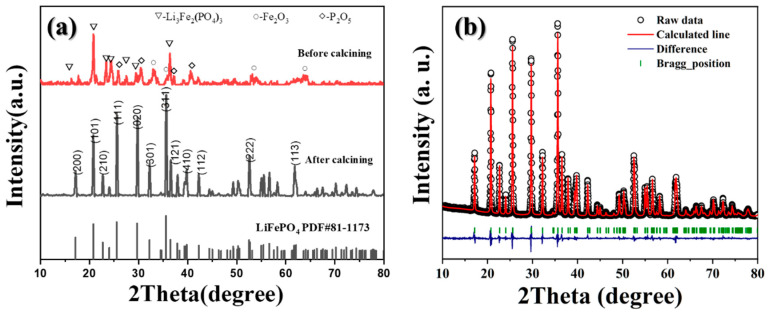
(**a**) XRD patterns of sample U_1.6_S_0.8_ before and after calcining. (**b**) Rietveld refinement of XRD patterns of U_1.6_S_0.8_.

The XRD patterns of the powders before and after calcining were presented in Figure 3a. Before calcining, the XRD was noisy due to its poor crystallinity and the powders were composed of phases such as Li_3_Fe_2_(PO_4_)_3_ (JCPDS Card No. 47-0107), Fe_2_O_3_ (JCPDS Card No. 85-0987), and P_2_O_5_ (JCPDS Card No. 23-1301). During the high-temperature calcining process in the N_2_, reducible activated carbon was generated from the pyrolysis process of glucose. And Fe (III) phases were subsequently reduced and reacted to form LiFePO_4_ with Fe (II) as per the following possible reactions. As a result, LiFePO_4_/C with carbon coating was produced.

(4) $$4{\mathrm{Li}}_{3}{\mathrm{Fe}}_{2}({\mathrm{PO}}_{4}{)}_{3}+2{\mathrm{Fe}}_{2}O_{3}+3C\to 12{\mathrm{LiFePO}}_{4}+3CO_{2}$$

(5) $$2Fe_{2}O_{3}+2Li_{2}O+2P_{2}O_{5}^{}+C\to 4LiFePO_{4}+CO_{2}$$

Figure 3b presents the Rietveld refinement results for the U_1.6_S_0.8_ sample. All reflections were successfully indexed based on the orthorhombic LiFePO_4_ crystal structure within the Pnma space group, and no impurities were detected. Furthermore, a carbon phase was not found for its amorphous structure. The obtained lattice parameters (a = 10.331 Å, b = 6.008 Å, and c = 4.694 Å) are consistent with those reported in reference [28]. Figure S1 illustrates the XRD patterns of Nano-LiFePO_4_/C samples after being calcined at 750 °C for 6 h. All the diffraction peaks of the samples can be indexed according to orthorhombic crystal structure and no obvious impurity phases were observed, suggesting the successful preparation of single-phase LiFePO_4_/C (JCPDS Card No. 81-1173). Carbon content testing was conducted using a carbon and sulfur combined tester, and the results are presented in Table 1. The carbon in the Nano-LiFePO_4_/C composite material originates from two sources: (1) 20 wt% glucose added to the as-combusted powders before calcination and (2) residual impurities containing carbon from the excess urea–sorbitol mixed fuels. Figure S2 illustrates the thermogravimetric curve of urea–sorbitol mixed fuels in an air atmosphere. The mixed fuel initiates decomposition at approximately 200 °C, with 10.6% and 4.1% residue remaining at 350 °C and 500 °C, respectively. In our study, an excess of fuels (*φ* > 1) was employed. Despite subjecting the materials to a further high-temperature treatment at 500 °C, there was still organic content from excess fuel residues present in the LiFePO_4_/C samples. When *y* = 0.8, U_0.8_S_0.8_, U_1.2_S_0.8_, and U_1.6_S_0.8_ exhibited a relatively low carbon content, each with less than 3 wt%, indicating that the urea content had no noticeable impact on the carbon content as the excess urea pyrolyzed at high temperatures. Conversely, with *x* = 4, the samples displayed an increased carbon content, corresponding to the rising residual content resulting from the increased usage of mixed fuels.

**Figure 4:**
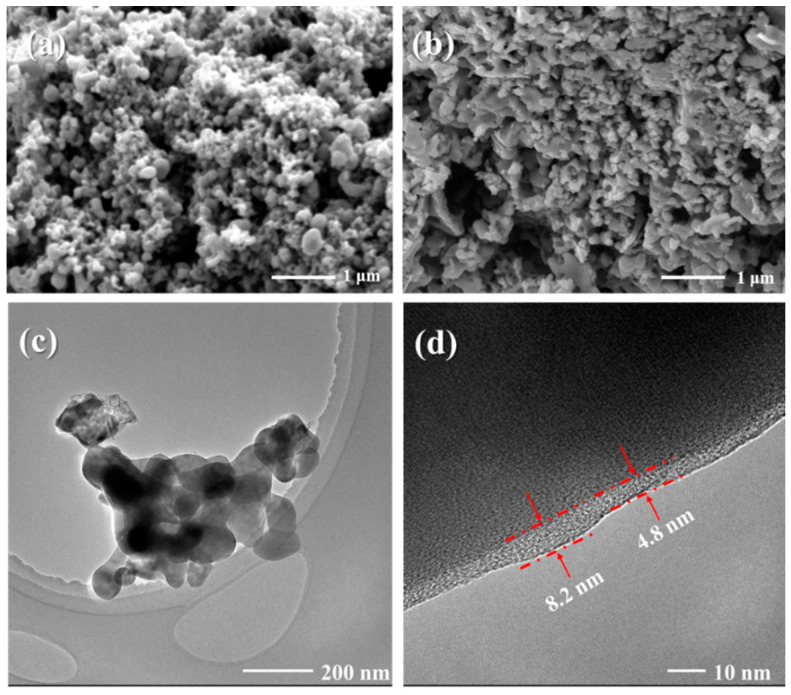
SEM images of sample U_1.6_S_0.8_ (**a**) before calcining and (**b**) after calcining. (**c**,**d**) HRTEM images of U_1.6_S_0.8_.

**Figure 5:**
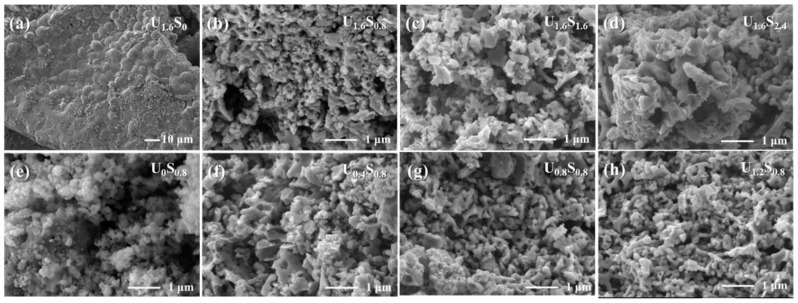
SEM images of LiFePO_4_/C powders prepared using different usage of fuels. (**a**) U_1.6_S_0_; (**b**) U_1.6_S_0.8_; (**c**) U_1.6_S_1.6_; (**d**) U_1.6_S_2.4_; (**e**) U_0_S_0.8_; (**f**) U_0.4_S_0.8_; (**g**) U_0.8_S_0.8_; (**h**) U_1.2_S_0.8_.

The SEM images of sample U_1.6_S_0.8_ before and after calcination are shown in Figure 4a,b, respectively. Before calcining, a highly porous structure could be observed and the precursor exhibited well-distributed spherical particles with a mean particle size of less than 100 nm. The release of numerous gases and the rapid reaction rate during the combustion process impede particle growth, resulting in the nanoparticles and the formation of pores within the resulting powder. After calcining at 750 °C, an obvious increase in particle size was observed due to particle sintering and growth at high temperatures. SEM images of Nano-LiFePO_4_/C samples with different fuel amounts are presented in Figure 5. As the combustion reaction had not occurred, the sample prepared only using urea showed a bulky and dense microstructure. For other powders prepared with different content of fuels, they showed a porous and foamy structure as a result of the large amounts of gases released during the combustion reaction. The maximum temperature (*T_m_*) and mean particle size of the samples are summarized in Table 2. With the increasing amount of sorbitol, the reaction between the nitrate and the fuels released more heat, resulting in a higher *T_m_*. The particle distribution curves are shown in Figure S3. Consequently, the particle size increased with the amount of sorbitol for particle growth and sintering at higher temperatures. As a contrast, particle size as well as T_m_ decreased with the amount of urea because a higher level of gas release effectively reduced the maximum temperature and aggregation. Figure 4c,d present the HRTEM images of U_1.6_S_0.8_. Aggregation with particles of a size less than 150 nm could be observed. Nano-LiFePO_4_/C particles were covered by continuous carbon layers, which could improve the surface stability and enhance the conductivity between particles [29].

**Figure 6:**
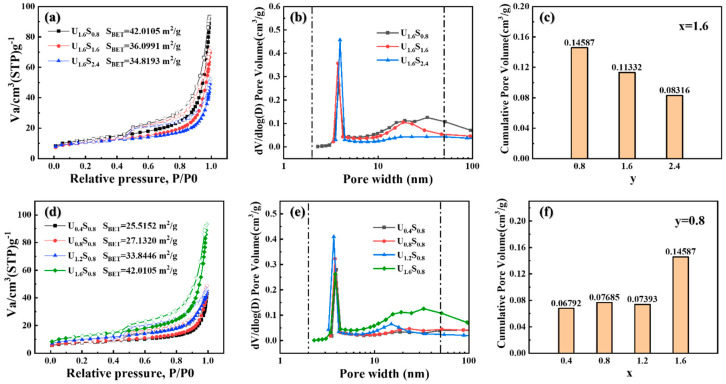
Nitrogen adsorption–desorption isotherms and pore size distribution of (**a**–**c**) U_1.6_S*_y_* and (**d**–**f**) U*_x_*S_0.8_.

The N_2_ adsorption–desorption isotherms and pore size distribution plots of samples prepared using mixed fuels are presented in Figure 6. The isotherm curves show H3 hysteresis on IV type isotherms with foamy microstructure [30] and the samples possess a mesopore nature distributed in the range of 2–50 nm. Samples prepared using SCS showed a high specific surface area and a highly porous structure. It is clearly demonstrated that the surface area and pore volume varied with the amount of urea/sorbitol. As the content of sorbitol increased, both the specific surface area and pore volume decreased due to the higher *T_m_* and larger particle size. On the other hand, the specific surface area and pore volume tended to increase with the content of urea, which was attributed to the higher amount of gas released and the smaller particle size. As a result, sample U_1.6_S_0.8_ showed the highest specific surface area (42.01 m^2^/g) and pore volume (0.146 cm^3^/g) due to its lowest *T_m_* and smallest particle size.

**Figure 7:**
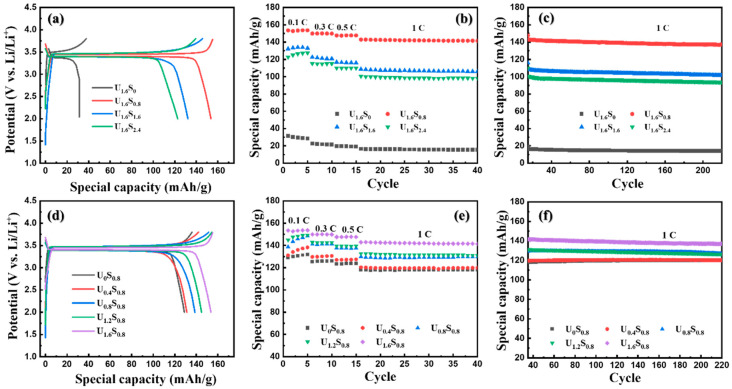
(**a**,**d**) Charge–discharge voltage profiles, (**b**,**e**) rate performance and (**c**,**f**) cycling performance at 1 C of U_1.6_S_y_ and U_x_S_0.8_.

The initial charge–discharge curves of samples at a rate of 0.1 C s are compared in Figure 7a,d with the voltage range of 2.0 to 3.8V. All profiles displayed a similar smooth and monotonous charge–discharge voltage ramp with a plateau of around 3.4 V owing to the phase transition between LiFePO_4_ and FePO_4_. Sample U_1.6_S_0_ delivered the lowest discharge capacities for its bulky microstructure. For sample U_1.6_S*_y_*, they showed discharge capacities of 153.5, 132.2, and 122.7 mAh/g, respectively, which decreased with the increasing content of sorbitol. The deterioration of capacity was attributed to the increasing particle size resulting from increasing the *T_m_* [12]. For sample U*_x_*S_0.8_, samples showed discharge capacities of 128.9, 131.2, 138.7, 144.9, and 153.5 mAh/g, respectively. The discharge capacities of the samples increased with the content of urea due to the reduction in particle size caused by decreased the T_m_. The rate performance of samples is displayed in Figure 7b,e. Additionally, as shown in Figure 7c,f, except for U_1.6_S_0_, samples prepared using SCS with urea–sorbitol mixed fuels exhibited a very stable discharge capacity with more than 93% capacity retention after 200 cycles at a relative high rate of 1 C. As summarized in Table 3, sample U_1.6_S_0.8_ with the least sorbitol and the most urea showed the highest capacity, which was 153.5, 149.9, 147.6, and 142.9 mAh/g at 0.1, 0.3, 0.5, and 1 C, respectively. This enhanced performance can be attributed to several key factors: 1. An increased electrolyte contact area: a larger particle surface area facilitates more extensive contact between the Nano-LiFePO_4_/C and the electrolyte, leading to an improved electrode–electrolyte interface, which promotes an efficient charge transfer [31]. 2. Enhanced lithium utilization: smaller particle sizes allow a higher proportion of the LiFePO_4_ material to actively participate in electrochemical reactions. This greater lithium utilization contributes to increased energy storage capacity and enhanced overall performance [32]. 3. Improved cycling stability: smaller particles are less susceptible to mechanical stress during the insertion and extraction of lithium ions. This enhanced mechanical durability results in improved cycling stability and a longer cycle life for the battery [33]. The morphological changes in the Nano-LiFePO_4_/C electrode were examined through SEM before and after undergoing 1 C cycling. As depicted in Figure S4, prior to cycling, the electrode exhibited a compact and relatively flat surface. However, after 220 cycles of cycling at 1 C, the electrode’s surface became irregular and displayed discernible cracks. These changes were attributed to electrolyte erosion and the expansion/contraction of particles during the Li^+^ intercalation and de-intercalation processes.

**Figure 8:**
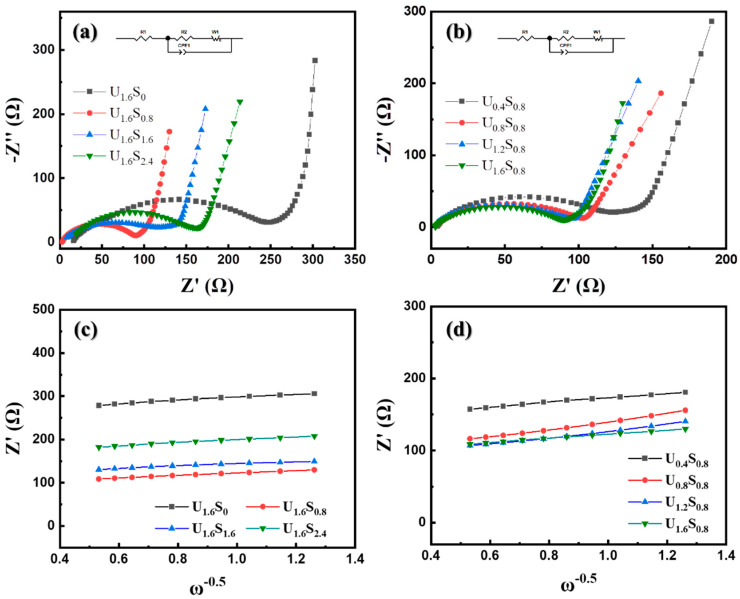
(**a**,**b**) Nyquist plots and (**c**,**d**) Z′ vs. ω^−0.5^ plots of U_1.6_S*_y_* and U*_x_*S_0.8_.

The resistances of the U_1.6_S*_y_* and the U*_x_*S_0.8_ electrodes before cycling were assessed and the resulting electrochemical impedance spectra (EIS) are displayed as Nyquist plots in Figure 8 in the frequency range from 0.1 Hz to 100 kHz. All EIS profiles present a semicircular pattern in the high-frequency region and an inclined line in the low-frequency region. In accordance with the equivalent circuit model, the semicircle in the high-frequency region is related to the charge transfer resistance (R_ct_). In the case of U_1.6_S*_y_*, the U_1.6_S_0_ sample showed the highest R_ct_ (242.4 Ω) due to its bulky microstructure. As y increased from 0.8 to 2.4, R_ct_ decreased from 139.3 Ω to 79.35 Ω, which is attributed to the reduction in particle size. For U*_x_*S_0.8_, R_ct_ exhibited a monotonic decrease as the quantity of urea used increased, which was primarily driven by the particle size reduction. The variations in R_ct_ offer a comprehensive explanation for the distinctions in electrochemical performance, indicating the favorable impact of a reduced particle size and a lower *T_m_* on the kinetics of Li^+^ ion charge transfer. The electronic conductivity of the samples was exhibited in Table S1. When *y* = 0.8, the conductivity of the powders increased with the usage of urea due to their decreasing particle size. U_1.6_S_1.6_ and U_1.6_S_2.4_ exhibited lower resistivity, which was attributed to their higher carbon content. The diffusion coefficient of Li^+^ ions was calculated using the Warburg factor (*σ*) as shown below [34]:

(6) $$D_{{\mathrm{Li}}^{+}}=R^{2}T^{2}{/}^{2}A^{2}n^{4}F^{4}C^{2}\sigma^{2}$$

where *R* is the gas constant, *T* is the absolute temperature, *A* is the surface area of the electrode, *n* is the number of electrons, *F* is Faraday’s constant, *C* is the concentration of the lithium ions, and *σ* is the Warburg factor. The calculation of D_Li+_ was listed in Table S2. D_Li+_ tended to increase with the decrease in particle size and U_1.6_S_0.8_ showed the highest D_Li+_. This result further illustrates that the particle size has a direct relationship with the Li^+^ transport. A small particle size could facilitate the transport of Li^+^, leading to the improvement of electrochemical performance.

**Figure 9:**
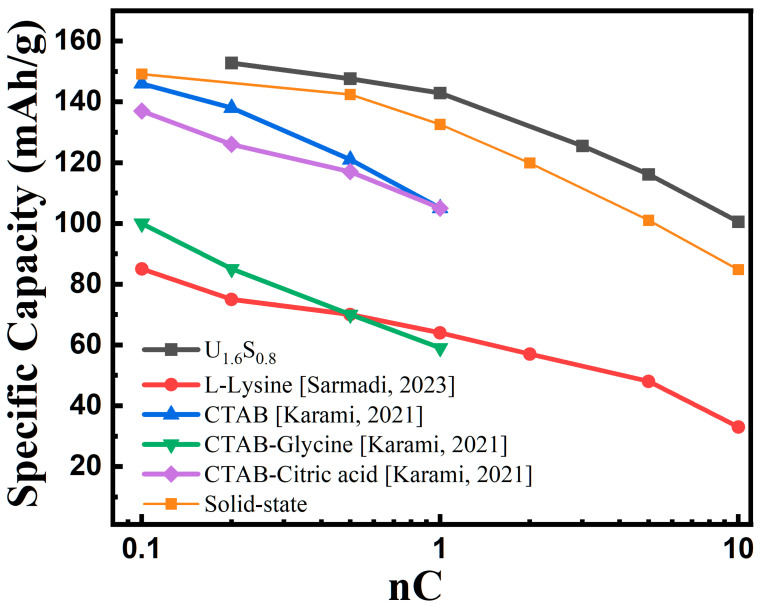
Comparison of discharge capacity at various rates of U_1.6_S_0.8_ sample with samples prepared using SCS in existed reports and solid-state method [19,21,22].

To assess the performance of Nano-LiFePO_4_/C synthesized using a combination of urea and sorbitol as mixed fuel, a comparative analysis of the electrochemical performance of sample U_1.6_S_0.8_ at various discharge rates was conducted. The rate performance of U_1.6_S_0.8_ at 0.2, 1.5, 1, 3, 5, and 10 C was shown in Figure S5. We compared this sample with SCS samples prepared using alternative fuel in the existing literature. As displayed in Figure 9, U_1.6_S_0.8_ exhibits the highest discharge capacity when subjected to discharge rates ranging from 0.2 C to 10 C, surpassing the performance of samples prepared with L-Lysine and CTAB-based fuels. Notably, U_1.6_S_0.8_ displays the least significant decrease in specific capacity as the discharge rate escalates from 0.2 C to 10 C, which is attributed to its diminutive particle size and high specific surface area. In comparison to commercial LiFePO_4_/C synthesized through a solid-state method, samples produced via SCS with urea–sorbitol mixed fuels demonstrate a competitive electrochemical performance, showing strong potential for commercial applications in the future.

## 4. Conclusions

Single-phase carbon coated Nano-LiFePO_4_/C was prepared using a solution combustion reaction with inexpensive urea–sorbitol mixed fuels. The content and ratio of sorbitol and urea have a significant influence on its combustion behavior and microstructure as well as on its electrochemical performance. The *T_m_* increases with an increased amount of sorbitol, resulting in a larger particle size, a lower specific surface area, and decreased specific capacity. In contrast, *T_m_* decreases with an increased amount of urea leading to a smaller particle size, a higher specific surface area, and improved electrochemical performance. Therefore, U_1.6_S_0.8_ exhibits attractive electrochemical performance with 153.5, 149.9, 147.6, and 142.9 mAh/g at 0.1, 0.3, 0.5, and 1 C owing to it having the smallest particle size and largest specific area. And it can retain more than 98% of the initial reversible capacity after 200 cycles at a rate of 1 C. This study could provide technical support for the future application of Nano-LiFePO_4_/C prepared using SCS.

## Figures and Tables

**Table 1:** Carbon content of LiFePO_4_/C samples.

Sample	Carbon Content (wt%)
U_0.8_S_0.8_	2.65
U_1.2_S_0.8_	2.72
U_1.6_S_0.8_	2.21
U_1.6_S_1.6_	4.10
U_1.6_S_2.4_	5.54

**Table 2:** The maximum temperature and particle size of LiFePO_4_/C samples.

Sample	T_m_	Particle Size/nm	Sample	T_m_	Particle Size/nm
U_1.6_S_0_	-	≥3 μm	U_0.4_S_0.8_	529	167 ± 84
U_1.6_S_0.8_	459	103 ± 51	U_0.8_S_0.8_	495	132 ± 60
U_1.6_S_1.6_	516	131 ± 65	U_1.2_S_0.8_	474	121 ± 48
U_1.6_S_2.4_	551	168 ± 92	U_1.6_S_0.8_	459	103 ± 51

**Table 3:** Summary of rate performance, initial coulombic efficiency, and capacity retention after 200 cycles at 1 C.

Samples	Initial Discharge Capacity (mAh/g)				Initial Coulombic Efficiency/%	Capacity Retention (1 C for 200 Cycles)
	0.1 C	0.3 C	0.5 C	1 C		
U_0_S_0.8_	128.9	125.5	123.4	118.0	94.8	100%
U_0.4_S_0.8_	131.2	129.7	127.0	120.4	92.3	99.8%
U_0.8_S_0.8_	138.7	141	137.7	129.9	91.4	97.8%
U_1.2_S_0.8_	144.9	142.8	139.5	132.7	93.8	95.3%
U_1.6_S_0.8_	153.5	149.9	147.6	142.9	98.8	95.8%
U_1.6_S_1.6_	132.2	122.6	116.8	108.7	91.1	93.5%
U_1.6_S_2.4_	122.7	115.2	110.1	100.6	88.3	93.3%
U_1.6_S_0_	31.5	22.7	19.8	16.5	83	84.5%

## Data Availability

Date is available on request.

## Supplementary Materials

The following supporting information can be downloaded at: https://www.mdpi.com/article/10.3390/ma16227155/s1, Figure S1: XRD patterns of LiFePO_4_ powders; Figure S2: The TG curve of urea/sorbitol mixed fuels in air atmosphere; Figure S3: Particle distribution plots of Nano-LiFePO_4_/C samples; Figure S4: The morphology of U_1.6_S_0.8_ electrode (a,b) before and (c,d) after cycling at 1C for 220 cycles; Figure S5: The rate performance of Nano-LiFePO_4_/C samples; Table S1: The electronic conductivity of as-calcined LiFePO_4_/C samples; Table S2: Calculation of D_Li+_ of LiFePO_4_/C samples.
