# Supplementary material for: Solution Combustion Synthesis of High-Performance Nano-LiFePO4/C Cathode Material from Cost-Effective Mixed Fuels
Source: Materials (Basel). 2023 Nov 14;16(22):7155. doi: 10.3390/ma16227155 (PMC10672621; doi:10.3390/ma16227155)
Supplement: Supplementary file 1 [file materials-16-07155-s001.zip › materials-2689635-supplementary.pdf]

Results

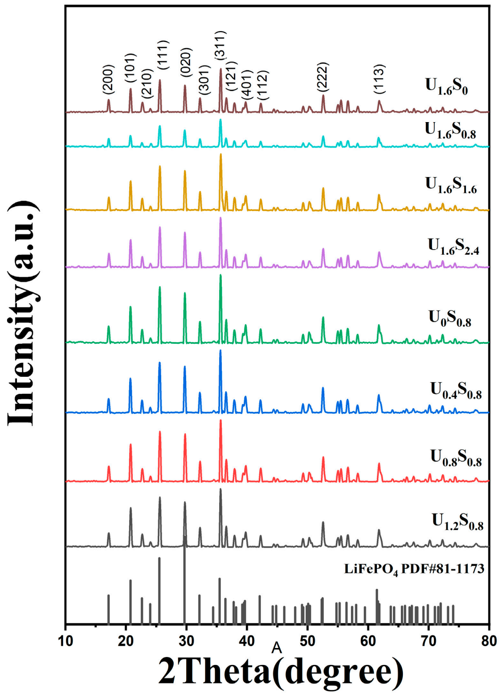

Figure S1. XRD patterns of $LiFePO_4$ powders.

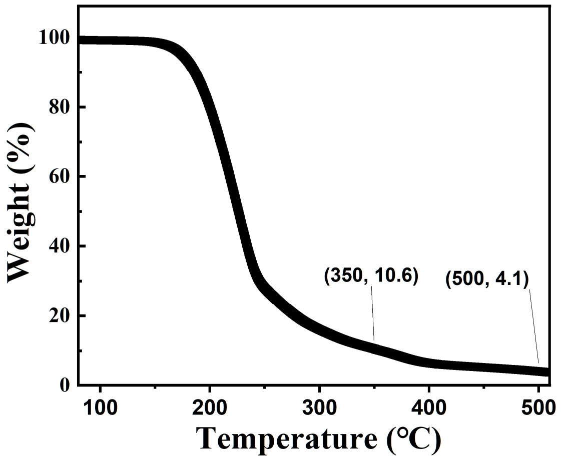

Figure S2. The TG curve of urea/sorbitol mixed fuels in air atmosphere.

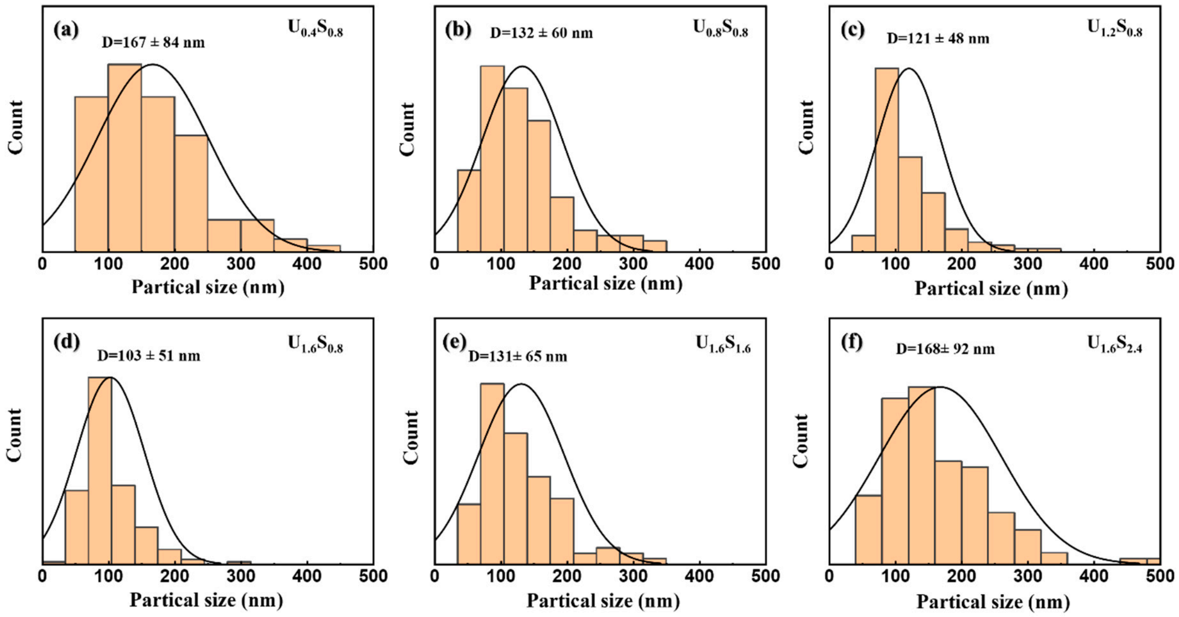

Figure S3. Particle distribution plots of Nano-$LiFePO_4$/C samples. (**a**) $U_{0.4}S_{0.8}$; (**b**) $U_{0.8}S_{0.8}$; (**c**) $U_{1.2}S_{0.8}$; (**d**) $U_{1.6}S_{0.8}$; (**e**) $U_{1.6}S_{1.6}$; (**f**) $U_{1.6}S_{2.4}$.

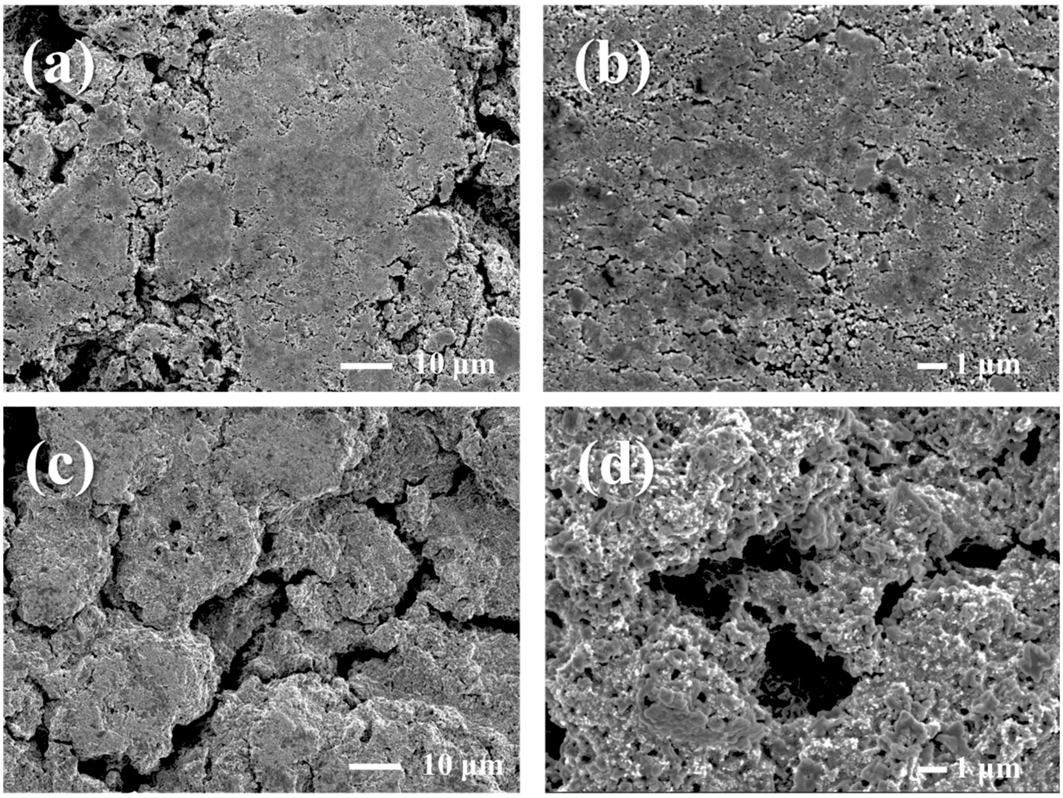

Figure S4. The morphology of $U_{1.6}S_{0.8}$ electrode (**a**,**b**) before and (**c**,**d**) after cycling at 1C for 220 cycles.

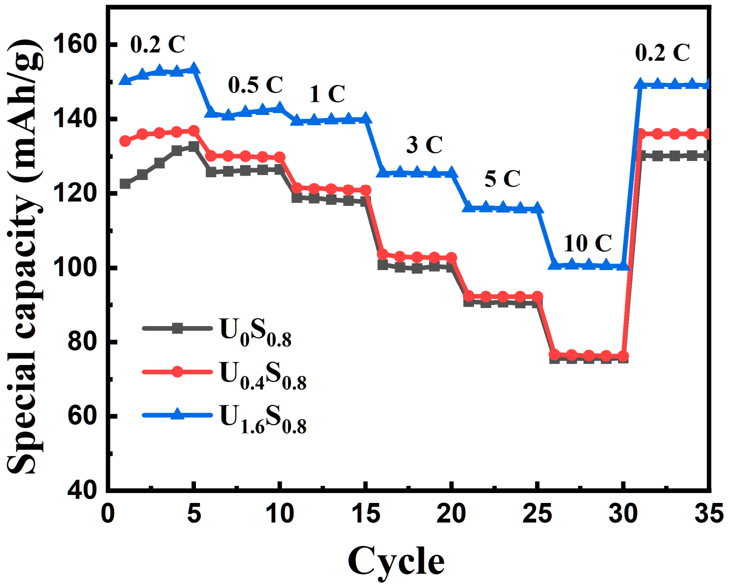

Figure S5. The rate performance of Nano-$LiFePO_4$/C samples.

Table S1. The electronic conductivity of as-calcined $LiFePO_4$/C samples.

**Sample**	**Resistivity/Ω·mm**	**Conductivity/S·mm^{-1}**
**$U_{0.4}S_{0.8}$**	1736	5.76×10^{-4}
**$U_{0.8}S_{0.8}$**	988	1.01×10^{-3}
**$U_{1.2}S_{0.8}$**	591	1.69×10^{-3}
**$U_{1.6}S_{0.8}$**	137	7.30×10^{-3}
**$U_{1.6}S_{1.6}$**	87	1.15×10^{-2}
**$U_{1.6}S_{2.4}$**	95	10.510^{-2}

Table S2. Calculation of D_{Li+} of $LiFePO_4$/C samples.

**Samples**	**σ/ΩS$^{-0.5}$**	**D_{Li+}/cm^{2}s^{-1}**
$U_{0.4}S_{0.8}$	35	4.60073E-14
$U_{0.8}S_{0.8}$	53	2.00637E-14
$U_{1.2}S_{0.8}$	45	2.78316E-14
$U_{1.6}S_{0.8}$	28	7.18864E-14
$U_{1.6}S_0$	37	4.11679E-14
$U_{1.6}S_{1.6}$	29	6.84225E-14
$U_{1.6}S_{2.4}$	30	6.2621E-14
